# Physical inactivity and smoking after myocardial infarction as predictors for readmission and survival: results from the SWEDEHEART-registry

**DOI:** 10.1007/s00392-018-1360-x

**Published:** 2018-08-23

**Authors:** Amanda Ek, Örjan Ekblom, Kristina Hambraeus, Åsa Cider, Lena V. Kallings, Mats Börjesson

**Affiliations:** 1 The Åstrand Laboratory of Work Physiology, The Swedish School of Sport and Health Sciences, P.O. Box 5626, 11486 Stockholm, Sweden; 2 Functional Area Occupational Therapy and Physiotherapy, Allied Health Professionals Function, Karolinska University Hospital, Stockholm, Sweden; 3 Department of Cardiology, Falun Hospital, Falun, Sweden; 4 Department of Neuroscience and Physiology, Gothenburg University, Gothenburg, Sweden; 5 Sahlgrenska University Hospital, Gothenburg, Sweden; 6 Department of Public Health and Caring Sciences, Family Medicine and Preventive Medicine, Uppsala University, Uppsala, Sweden; 7 Department of Food, Nutrition and Sports Science, Gothenburg University, Gothenburg, Sweden

**Keywords:** Myocardial ischaemia, Physical activity, Tobacco, Survival, Hospitalisation

## Abstract

**Background:**

Physical activity (PA) and smoking cessation are included in the secondary prevention guidelines after myocardial infarction (MI), but they are still underutilised. This study aims to explore how PA level and smoking status (6–10 weeks post-MI) were associated with 1-year readmission and mortality during full follow-up time, and with the cumulative 5-year mortality.

**Methods:**

A population-based cohort of all hospitals providing MI-care in Sweden (SWEDEHEART-registry) in 2004–2014. PA was expressed as the number of exercise sessions of ≥ 30 min in the last 7 days: 0–1 (low), 2–4 (medium) and 5–7 (high) sessions/week. Individuals were categorised as smokers, former smokers or never-smokers. The associations were analysed by unadjusted and adjusted logistic and Cox regressions.

**Results:**

During follow-up (*M* = 3.58 years), a total of 1702 deaths occurred among 30 644 individuals (14.1 cases per 1000 person-years). For medium and high PA, the hazard ratios (HRs) for mortality were 0.39 and 0.36, respectively, compared with low PA. For never-smokers, the HR was 0.45 and former smokers 0.56 compared with smokers. Compared with low PA, the odds ratios (ORs) for readmission in medium PA were 0.65 and 0.59 for CVD and non-CVD causes, respectively. For high PA, the corresponding ORs were 0.63 and 0.55. The association remained in adjusted models. There were no associations between smoking status and readmission.

**Conclusions:**

The PA level and smoking status are strong predictors of mortality post-MI and the PA level also predicts readmission, highlighting the importance of adherence to the secondary prevention guidelines.

**Electronic supplementary material:**

The online version of this article (10.1007/s00392-018-1360-x) contains supplementary material, which is available to authorized users.

## Introduction

Ischaemic heart disease (IHD) is a progressive disease and patients with established IHD run an increased risk of new cardiac events and subsequent death [1]. Both physical activity (PA) counselling and smoking cessation are included in the secondary prevention recommendations [2, 3]. However, not all individuals choose to participate in, or are even offered exercise-based cardiac rehabilitation, post-myocardial infarction (MI). Regarding smokers, almost 50% continued smoking 1 year post-MI [4].

In primary prevention, the self-reported level of PA and smoking cessation have been shown to predict future morbidity and mortality [3, 5, 6]. One previous study including 39 countries concluded that, individuals with IHD and a higher PA level have low risk of all-cause mortality [7].

Specifically in MI survivors, previous smaller studies have indicated a lower risk of all-cause mortality among the more physically active [8, 9]. For readmission, different studies showed conflicting results regarding the association between PA level and readmission due to MI among individuals with IHD [7, 8, 10]. Nevertheless, none of these studies looked at risk of readmission to hospital in general, e.g. due to cardio-vascular disease (CVD) or non-CVD. After an MI, most studies have been performed within the hospital setting focusing on exercise-based cardiac rehabilitation to increase physical fitness, and have therefore not included the physical activities performed outside the setting of cardiac rehabilitation.

For smoking, different studies show conflicting results regarding the association between smoking and the risk of new cardiac events post-MI, but smoking cessation has been found, in long-term follow-up, to reduce the mortality risk [10–14].

The importance of the post-MI PA level and smoking status for the prediction of future events merits further study, especially in larger coherent cohorts, with relevant follow-up. Thus, the primary aim of this large nationwide cohort study, including a majority of all MI survivors in Sweden over a period of 10 years, was to explore how the post-MI PA level and smoking habits were associated with 1-year readmission to hospital, as well as with all-cause mortality during the long-term follow-up (assessed as the mortality over the full follow-up time as well as the 5-year mortality).

## Methods

This was a national cohort study, including data from the SWEDEHEART registry between the years 2004 and 2014. SWEDEHEART is an on-going quality registry for inpatient and outpatient cardiac care including different sub-registers. The RIKS-HIA sub-register includes information about the quality of inpatient cardiac care in patients with MI. The SEPHIA sub-register includes outpatient follow-up. Importantly, as all Swedish citizens have a personal identification number, complete follow-up of clinical events can be obtained via linkage to the Swedish National Population Register [15].

### Study population

**Fig. 1:**
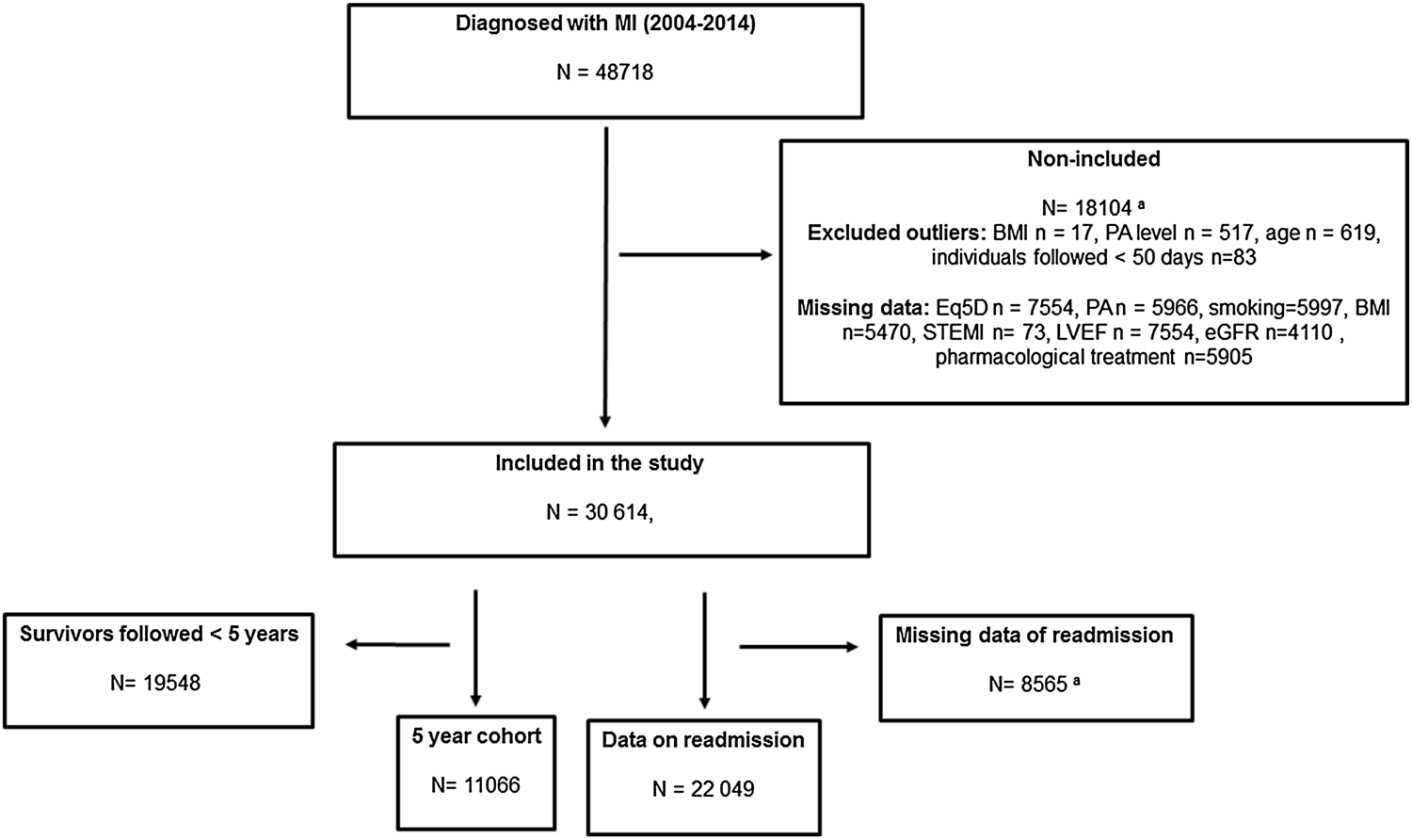
Flowchart of recruitment of study population. ^a^Excluded because of one or several outliers or missing data

Individuals included in the study were patients aged 18–74 diagnosed with their first MI, coded as I.21 according to the International Classification of Diseases, 10th revision (ICD 10). In addition, complete data on covariates and explanatory and outcome variables (as outlined below) should be available for inclusion. Individuals excluded from the standardised 5-year follow-up were survivors with less than 5 years of follow-up (Fig. 1).

### Data

The primary outcome measures were mortality and readmission. Mortality was measured in two ways, first as 5-year mortality and then as the full survival time after MI, data on which were collected from the Swedish National Population Register.

Data on readmission to hospital during the first year post-MI were obtained from the SEPHIA registry and collected by patient interviews and reviews of hospital records. Readmissions were grouped into cardiovascular disease (CVD); i.e. angina pectoris, heart failure (HF), MI, or stroke, or readmission due to non-CVD diseases.

Explanatory variables were self-reported smoking status and PA level 6–10 weeks after the MI from the SEPHIA registry. PA level was assessed using a 7-day recall question: “Number of exercise sessions of at least 30 min (two 15-min sessions can be combined into a 30-min session) in the last 7 days, with an intensity of at least fast walking”. Based on the answers, patients were then grouped into three categories: 0–1 sessions/week = low activity, 2–4 sessions/week = medium activity and 5–7 sessions/week = high activity. The highest category corresponds to the internationally recommended level of PA for health, i.e. at least 150 min of at PA/week, of at least moderate intensity. This question was specifically constructed for the SEPHIA registry and has not previously been tested for reliability and validity. Smoking status was classified as: never-smokers, former smokers (no smoking during the last month) and current smokers.

Covariates included were age, gender, ST elevation MI (STEMI), left ventricular ejection function (LVEF), body mass index (BMI), percutaneous coronary intervention (PCI), health-related quality of life (HQoL), estimated glomerular filtration rate (eGFR) and full pharmacological treatment, since they may all affect readmission and mortality [2, 16–22]. HQoL was measured with the validated HQoL instrument EuroQol5D (EQ5D) 6–10 weeks post-MI [23]. The EQ5D values were converted into a single summary index, ranging from − 0.594 to 1, where 1 represents the best possible health [24]. The mean score in a Swedish population has been reported to be 0.84 [25]. Hence, patients were assigned to two groups based on this value (≤ 0.84 and > 0.84, respectively). LVEF was categorised into four groups: normal (LVEF > 50%), mild impairment (LVEF 40–50%), moderate impairment (LVEF 30–39%), and severe impairment (LVEF < 30%). eGFR was based on serum creatinine values (sCr) calculated according to the Cockcroft–Gault formula [16]. In order to separate normal or mildly decreased eGFR from moderately decreased or more pronouncedly decreased eGFR, eGFR was dichotomised at 60 mL/min/1.73 m^2^. Achievement of full pharmacological treatment, i.e. being treated with ACE-inhibitors, beta-blocking agent, statins or other lipid-lowering agents and antithrombotic agents were dichotomised into yes and no, for the further analyses.

BMI and age at the incidence of the MI were treated as continuous non-parametric variables.

### Statistical analyses

Categorical variables are presented as frequencies and relative frequencies, whereas continuous non-parametric variables are presented as medians with interquartile ranges (IQRs). Differences between included and excluded individuals (gender, age) and baseline variables (explanatory variables and all covariates) were examined using the Chi-square test for categorical variables and the Mann–Whitney *U* test for continuous variables. Cox regressions were performed to explore any differences in time to event (mortality) between patients according to PA level and smoking status categories. Results from the Cox regressions were presented as hazard ratios (HRs) with 95% confidence intervals (CIs). The first regression model was an unadjusted model, the second included age and gender and the third included all covariates.

The attributable risk (AR) of mortality in different PA categories or smoking status categories was calculated by subtracting the incidence of unexposed (never-smoker and former smoker or medium PA level and high PA level, respectively) from the incidence of exposed (smoker and low PA level) patients. The attributable fraction (AF) was obtained by dividing the AR by the incidence of exposed patients.

In order to explore the association between PA level, smoking status and risk of readmission during the first post-MI year, logistic regressions were used with the same three models as in the Cox regressions. Results from the logistic regressions were presented as odds ratios (ORs), with 95% CIs. Differences in HR and OR were analysed as interactions based on Altman et al. [26]. All statistical analyses were performed using the SPSS 24.0 software (IBM Corp., Armonk, NY, USA).

## Results

### Baseline characteristics

A total of 48,718 patients were treated for MI in the Swedish coronary care units and included in the SWEDEHEART register during the study period. Complete data were found for 30 614 unique individuals and these were included in the study. Figure 1 shows a flowchart of the recruitment of the study population. Half of the group stated that they performed PA at high levels (5–7 sessions/week), and one of three were never-smokers (Table 1).

**Table 1:** Patient baseline characteristics by survivors and non-survivors

	All	Survivors	Non-survivors	*p* value^i^
	*N* = 30,614 (%)	*N* = 28,912 (%)	*N* = 1702 (%)	
Gender, male	22,608 (74)	21,431 (74)	1177 (69)	< 0.001
Age, years	63 (IQR 12)	63 (IQR 12)	67 (IQR 9)	< 0.001
STEMI^a^	12,995 (42)	12,370 (43)	625 (37)	< 0.001
PCI^b^	24,416 (80)	23,292 (81)	1124 (66)	< 0.001
LVEF^c^				< 0.001
> 50%	20,394 (67)	19,529 (68)	865 (51)	
40–49%	6176 (20)	5784 (20)	392 (23)	
30–39%	3192 (10)	2875 (10)	317 (19)	
< 30%	852 (3)	724 (3)	128 (8)	
Body mass index (kg/m^2^)	27 (IQR 5)	27 (IQR 5)	26 (IQR 6)	< 0.001
Physical activity level^d^				< 0.001
Low	6434 (21)	5745 (20)	689 (41)	
Medium	8815 (29)	8433 (29)	382 (22)	
High	15,365 (50)	14,734 (52)	631 (37)	
Smoking status				< 0.001
Never-smokers	9849 (32)	9421 (33)	428 (25)	
Former smokers^e^	17 183 (56)	16,243 (56)	940 (55)	
Smokers	3582 (12)	3248 (11)	334 (20)	
HQoL^f^, EQ5D	0.85 (IQR 0.27)	0.85 (IQR 0.27)	0.80 (IQR 0.34)	< 0.001
eGFR^g^ <60 mL/min/1.73 m^2^	28,174 (92)	26,865 (93)	1309 (77)	< 0.001
Full pharmacological treatment^h^	21,300 (70)	20 173 (70)	1127 (66)	0.002

Data presented as numbers with percentages in brackets or median with IQR in brackets

^a^ST elevation myocardial infarction

^b^Percutaneous coronary intervention

^c^Left ventricular ejection fraction

^d^Physical activity level; low = 0–1 sessions/week; medium = 2–4 sessions/week and high = 5–7 sessions/week

^e^No smoking during the last month

^f^Health-related quality of life

^g^Estimated glomerular filtration rate

^h^ACE-inhibitors, beta-blocking agent, statins or other lipid-lowering agents and anti-thrombogenic agents

^i^Differences between survivors and non-survivors

There were very small, albeit statistically significant differences (*p* < 0.001) in terms of gender, age, LVEF, STEMI, PCI, HQoL, eGFR and full pharmacological treatment between the total study population and those without complete data (non-included) (Supplementary file 1).

A total of 11066 subjects remained in the standardised 5-year follow-up analysis (Fig. 1). There were no differences in gender, between non-included individuals and those in the 5-year follow-up; however all other covariates showed small differences (*p* < 0.05) (Supplementary file 1).

Complete data on readmission rates were available for 22049 individuals (Fig. 1). Individuals with complete data on readmissions differed (*p* < 0.01), compared to the individuals where complete data on readmissions were missing (Supplementary file 1).

### Mortality

In the total study population, the individuals were followed for a median duration of 3.58 years. A total of 1702 deaths occurred during the study period. All variables differed (*p* < 0.05) between non-survivors and survivors (Table 1).

The total risk time was 120443 person-years and the mortality rate was calculated to 14.1 cases per 1000 person-years. The AFs for patients with low PA levels, compared with patients with medium and high PA levels, were 60.8 and 64.0%, respectively. The AFs for smokers, compared with former and never-smokers were 43.4 and 55.5%, respectively.

For medium and high PA levels, the HR was decreased compared to low PA level (*p* < 0.001). Similar figures were found in the standardised 5-year follow-up group (*p* < 0.001). There was no significant difference in HR between medium and high PA levels. The mortality was also lower among non-smokers and former smokers compared to smokers, both in the total study population and in the 5-year follow-up group. The decreased risk remained in multivariate analyses (*p* < 0.001). The HR was lower in never-smokers compared to former smokers, with never-smokers having the lower risk (*p* < 0.05) (Table 2).

**Table 2:** Incidence (cases per 1000 person-years), unadjusted and adjusted hazard ratios (HR with 95% CIs) for post-MI mortality in a 5-year standardised cohort (754 deaths) and full-time analysis (1702 deaths) with different PA levels and smoking status

Variable	Incidence	Full-time analysis (*N* = 30 614)			5-year mortality (*N* = 11,066)		
		Unadjusted HR	Adjusted HR^a^	Adjusted HR^b^	Unadjusted HR	Adjusted HR^a^	Adjusted HR^b^
Physical activity level^c^							
Low	28.37	Referent	Referent	Referent	Referent	Referent	Referent
Medium	11.13	0.39 (0.35–0.45)*	0.42 (0.37–0.47)*	0.52 (0.46–0.60)*	0.40 (0.34–0.49)*	0.44 (0.36–0.53)*	0.55 (0.46–0.67)*
High	10.20	0.36 (0.32–0.40)*	0.37 (0.33–0.41)*	0.50 (0.45–0.56)*	0.34 (0.29–0.40)*	0.35 (0.30–0.42)*	0.50 (0.42–0.59)*
Smoking status							
Smokers	24.36	Referent	Referent	Referent	Referent	Referent	Referent
Former smokers^d^	13.79	0.56 (0.49–0.63)*	0.45 (0.40–0.51)*	0.50 (0.44–0.57)*	0.56 (0.46–0.67)*	0.56 (0.47–0.67)*	0.50 (0.42–0.61)*
Never-smokers	10.84	0.45 (0.39–0.52)*^†^	0.32 (0.28–0.37)*^†^	0.38 (0.33–0.44)*^†^	0.44 (0.36–0.55)*^†^	0.44 (0.36–0.55)*^†^	0.37 (0.29–0.46)*^†^

^†^Differences between (*p* < 0.05) former smokers and never-smokers

*Differences between reference group

^a^Adjusted for age and gender

^b^Further adjusted for ST elevation myocardial infarction, percutaneous coronary intervention, left ventricular ejection fraction, body mass index, health-related quality of life, estimated glomerular filtration rate and full pharmacological treatment

^c^Physical activity level; low = 0–1 sessions/week; medium = 2–4 sessions/week and high = 5–7 sessions/week

^d^No smoking during the last month

### Readmission

Patients with any hospital readmission had a lower eGFR, PA level, LVEF, and HQOL. They were more often women, older, fewer of them had undergone PCI and a higher proportion had a STEMI compared with patients with no readmission (all *p* < 0.05). During the first year post-MI, there were a total of 2556 individuals (11.6%) with one or several readmissions to hospital due to CVD. Of those, the majority of cases were due to angina pectoris (*n* = 1659), followed by MI (*n* = 496), HF (*n* = 384) and stroke (*n* = 166). There were 3224 (14.6%) patients with readmissions due to non-CVD.

The risk of readmission due to CVD-related causes was 35 and 37% lower, respectively, for medium and high PA levels (Table 3) compared to low PA. The risk of readmission due to non-CVD was 41 and 45% lower for the two groups, respectively, compared to low PA. The association remained after adjusted multiple logistic regressions (*p* < 0.001). On the other hand, a significant association between smoking status and readmission for either CVD or non-CVD could not be shown in unadjusted models. If anything, in the fully adjusted models never-smokers seem to have a slightly higher risk of readmission (*p* < 0.01) (Table 3). There were no differences in the estimated OR between medium and high PA levels or between former and never-smokers (Table 3).

**Table 3:** Unadjusted and adjusted odds ratios (OR with 95% CIs) of readmission to hospital due to CVD (*N* = 2556) and non-CVD diseases (*N* = 3224) the first year after an MI among individuals with different PA level and smoking status

	1-year readmission CVD *N* = 22,049			1-year readmission non-CVD *N* = 22,049		
Variable	Unadjusted OR	Adjusted OR^a^	Adjusted OR^b^	Unadjusted OR	Adjusted OR^a^	Adjusted OR^b^
Physical activity level^c^						
Low	1.0 referent	1.0 referent	1.0 referent	1.0 referent	1.0 referent	1.0 referent
Medium	0.65 (0.58–0.73)***	0.65 (0.58–0.76)***	0.74 (0.66–0.83)***	0.59 (0.50–0.69)***	0.59 (0.50–0.69)***	0.65 (0.55–0.76)***
High	0.63 (0.57–0.70)***	0.64 (0.58–0.71)***	0.75 (0.68–0.84)***	0.55 (0.47–0.63)***	0.56 (0.48–0.64) ***	0.63 (0.55–0.73)***
Smoking status						
Smokers	1.0 referent	1.0 referent	1.0 referent	1.0 referent	1.0 referent	1.0 referent
Former smokers^d^	1.07 (0.93–1.22)	1.10 (0.96–1.26)	1.15 (0.99–1.34)	1.02 (0.84–1.24)	1.08 (0.88–1.31)	1.18 (0.97–1.44)
Never-smokers	0.98 (0.85–1.13)	1.00 (0.87–1.16)	1.20 (1.04–1.38)	1.10 (0.89–1.35)	1.16 (0.94–1.43)	1.34 (1.09–1.66)**

^a^Adjusted for age and gender

^b^Further adjusted for ST elevation myocardial infarction, percutaneous coronary intervention, left ventricular ejection fraction, body mass index, health-related quality of life, estimated glomerular filtration rate and full pharmacological treatment

^c^Physical activity level; low = 0–1 sessions/week; medium = 2–4 sessions/week and high = 5–7 sessions/week

^d^No smoking during the last month

**Difference between smokers and never-smokers *p* < 0.01

***Difference (*p* < 0.001) between low and medium PA level, respectively, low and high PA level

## Discussion

**Fig. 2:**
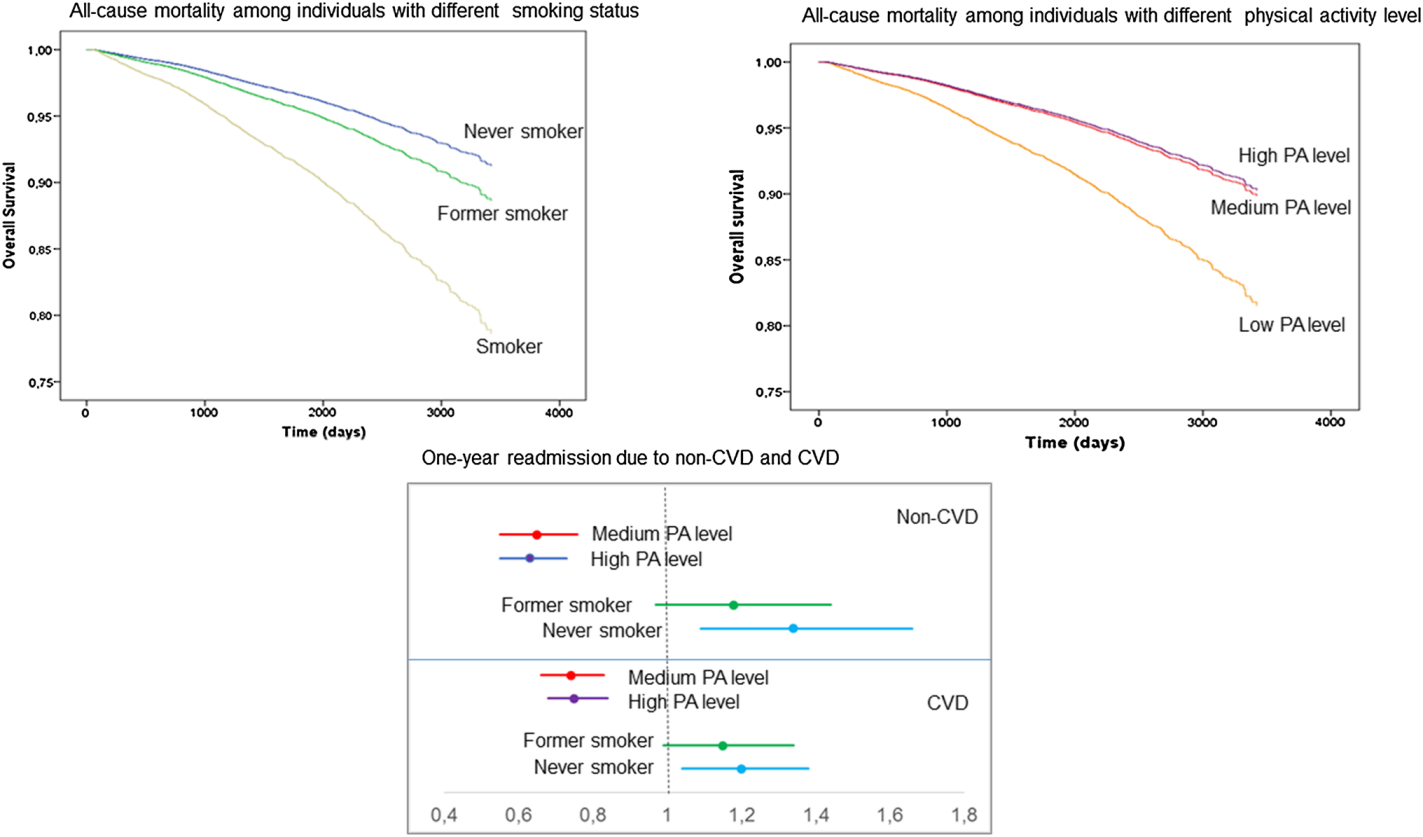
In fully adjusted analyses of the total study population both the PA level and smoking status are predictors of mortality post-MI. The PA level also predicts 1-year readmission

The main result of the present study is that self-reported PA level and smoking status both are predictors of all-cause mortality post-MI. In addition, PA level could predict the risk of readmission within 1 year (Fig. 2).

As far as we know, this is one of the first larger national cohort studies focusing on the association between lifestyle habits (i.e. PA level and smoking status) and hospital readmission and survival among patients post-MI. An important novelty of the study is that all individuals with complete data in the registers are included and that PA, regardless of whether it was performed within or outside of the health care setting, was taken into account. Earlier studies have mainly focused on exercise within the hospital setting, excluding more than half of all MI patients who do not participate in this type of activity [4].

In comparison with previous studies, the association with all-cause mortality in the present study appears to be even stronger [7–9]. The largest risk reduction for mortality was found between patients with a low PA level and those patients reporting a medium level of PA, and a less pronounced additional risk reduction was found among those patients with MI reporting a high PA level compared to medium PA. This is consistent with earlier studies including individuals with IHD as well as healthy individuals [7, 27], showing a non-linear relationship between PA levels and mortality. Thus, the greatest effect seems to be achieved by performing even a little physical activity [3, 5, 7].

The association between PA level and hospital readmission has to be evaluated in the context of earlier studies showing conflicting results [8, 10, 28]. However, to our knowledge, there are no other studies focusing on the overall PA level and readmissions for cardiovascular disease (CVD) and non-CVD disease, respectively, making the present study unique.

For smoking, the decreased risk of all-cause mortality was strongest for never-smokers. The association was weaker among former smokers, which may be due to that individuals who quit smoking at index of their MI were counted as former smokers. In addition, long-term effects of smoking contributing to endothelial dysfunction, may be existent in former smokers [29]. In contrast with earlier studies, there was no association in unadjusted analyses between smoking status and hospital readmission. However, previous studies had a longer follow-up time [11, 12], which may indicate that smoking status is a better predictor of long-term rather than short-term readmission and survival. Another reason might be that smokers developed CVD earlier (mean 4.0 years earlier, data not shown) than ex-smokers and never-smokers and therefore may have a lower burden of other risk factors. This is supported by the observation that smokers have a lower 1 year mortality [14], perhaps due to lower age. Although these potential confounders have been controlled for in our study, there is always a risk for residual confounding, which may be of importance in this analysis.

The pharmacological care in this study is similar in the group of survivors and in the non-surviving group, indicating that any difference observed could be attributable to other factors. From a clinical viewpoint, the results of the present study underline the importance of asking patients with MI about their PA level and of supporting the least active individuals to increase their level of PA. As both PA counselling and smoking cessation are important complement to pharmacological care and included in the secondary prevention guidelines, the results of the prognostic information given by the level of PA and smoking status may further improve efforts to implement such secondary preventive measures in the clinical setting. Despite lifestyle behaviour change being recommended as first-line treatment for secondary prevention, it is still underutilised [4]. Studies like this, may aid in the efforts to go from theory to practice, thereby contributing to equality in post-MI-care. As the greatest risk reduction seems to be associated with not being sedentary, achieving at least a low level of PA regularly, these results may help to implement lifestyle behavioural change, as a complement to exercise-based cardiac rehabilitation.

### Study limitations

This is a retrospective observational study exploring an association, and any conclusions regarding causality should be drawn with caution. Nevertheless, this large national cohort study includes a majority of the cardiac departments in Sweden, providing natural disease course data. A risk of reverse causality is present, so that individuals with a better health status may have a higher PA level. To reduce this risk, we adjusted for several known covariates [17–21, 23], most importantly those factors that are not associated to the level of PA (age, sex). However, there is always a possibility that other risk factors, not available in this study, could have impact on the results.

In order to be included in the survey, individuals had to have complete data of outcome and exploratory variables as well as all covariates, thereby excluding a high number of individuals. Nevertheless, including individuals with complete data decreases the risk of type II error and increases the internal validity. In addition, the groups with complete and incomplete data differed very little, albeit statistically significant, in terms of basal characteristics.

This study only includes patients surviving to the first SEPHIA follow-up (i.e. at 6–10 weeks), which may have contributed to the lower mortality rates, compared with other studies [30]. Information on readmissions during the first year was collected at the second follow-up in the SEPHIA (i.e. at 12–14 months). The smaller number of readmissions in this study may be due, in part, to individuals with severe disease or disabilities not being able to attend the second follow-up.

PA level and smoking status were surveyed using self-reported non-validated questions. The classification of the three PA categories was subjective. In clinical practice, self-reports are the most commonly used assessment technique. Questionnaires are an inexpensive and easily administered method [31]. In our study population a surprisingly high number of individuals reported a high level of PA. This might be explained by that, in comparison to objective measurement of PA, criterion validity of self-reported PA is limited [32]. This may be due to that it being difficult to estimate duration and intensity of PA, interpretation of the questions, as well as social desirability [31]. However, the predictive validity may well be comparable to objective measures [32]. In this study, the predictive validity for mortality and readmission was shown to be strong. In the future it would be of interest to assess the PA level among MI survivors with objective measurements such as accelerometers. For smoking status, the question explored current smoking status, but there was no information about duration and amount of smoking or previous habits. This may have affected the outcome.

## Conclusions

This national cohort study indicates that a higher level of PA is associated with a lower risk of 1-year hospital readmission and all-cause mortality post-MI. For non-smokers, there was a weaker association with lower all-cause mortality, and no association with 1-year readmission in unadjusted analyses. These results highlight the importance of asking patients with MI about their PA level and smoking status, as predictors of survival and readmission, in clinical practice. Most importantly, this information will further aid the implementation of the recommended secondary preventative measures [2] to increase the PA level and smoking cessation among patients after a MI. It is now time to go from recommendations to action, focusing on methods to increase PA in the clinical setting.

## Electronic supplementary material

Below is the link to the electronic supplementary material.

Supplementary material 1 (DOCX 15 KB)
