# Supplementary material for: Physical inactivity and smoking after myocardial infarction as predictors for readmission and survival: results from the SWEDEHEART-registry
Source: Clin Res Cardiol. 2018 Aug 23;108(3):324–32. doi: 10.1007/s00392-018-1360-x (PMC6394466; doi:10.1007/s00392-018-1360-x)
Supplement: Supplementary file 1 — Supplementary material 1 (DOCX 15 KB) [file 392_2018_1360_MOESM1_ESM.docx]

**Supplementary file 1.** Patient baseline characteristics by all individuals in the registers, total study population, five year cohort

and with complete data of readmission. Data presented as numbers with percentages in brackets or median with IQR in brackets

|  | | | **SWEDEHEART N= 48718^i^** | | | **Total study population N= 30614** | | **5 year cohort N=11066** | | **Readmission cohort N=22049** | |
| --- | --- | --- | --- | --- | --- | --- | --- | --- | --- | --- | --- |
| **Gender, male** | | | 35566 (73) | | | 22608 (74)*** | | 8140 (74) | | 16313 (74)*** | |
| **Age, years** | | | 63 (IQR 13) | | | 63 (IQR 12)*** | | 62 (IQR 12)*** | | 63 (13)*** | |
| **STEMI^a^** | | | 19874 (41) | | | 12995 (42)*** | | 4780 (43)*** | | 9391 (43)*** | |
| **PCI**^b^ | | | 37739 (78) | | | 24416 (79)*** | | 8394 (76)*** | | 17582 (80)*** | |
| **LVEF^c^** | | |  | | | *** | | * | | *** | |
| > 50% | | | 27035 (56) | | | 20394 (67) | | 7035 (64) | | 14749 (67) | |
| 40-49% | | | 8432 (17) | | | 6176 (20) | | 2413 (22) | | 4460 (20) | |
| 30-39% | | | 4429 (9) | | | 3192 (10) | | 1284 (12) | | 2274 (10) | |
| < 30% | | | 1268 (3) | | | 852 (3) | | 334 (3) | | 566 (3) | |
| **Body Mass Index**, kg/m^2^ | 27 (IQR 5) | | | | | 27 (IQR 5) | | 27 (IQR 5)*** | | 27 (IQR 5)** | |
| **Physical activity level**^d^ | | |  | | |  | | *** | | *** | |
| Low | | | 9161 (19) | | | 6434 (21) | | 2259 (20) | | 4275 (19) | |
| Medium | | | 11908 (24) | | | 8815 (29) | | 3077 (28) | | 6336 (29) | |
| High | | | 21166 (43) | | | 15365 (50) | | 5730 (52) | | 11438 (52) | |
| **Smoking status** | | |  | | |  | | ** | | *** | |
| Never smokers | | | 13859 (28) | | | 9849 (32) | | 3427 (31) | | 7200 (33) | |
| Former smokers^e^ | | | 23830 (49) | | | 17183 (56) | | 6325 (57) | | 12438 (56) | |
| Smokers | | | 5032 (10) | | | 3582 (12) | | 1314 (12) | | 2411 (11) | |
| **HQoL^f^,** EQ5D | | | 0.85 (IQR 0.27) | | | 0.85 (IQR 0.27)*** | | 0.85 (IQR 0.27)*** | | 0.73 (0.25)*** | |
| **eGFR^g^** <60 mL/min/1.73m^2^ | | | 3642 (8) | | | 2221 (7)*** | | 841 (8)* | | 1542 (7)*** | |
| **Full pharmacological treatment^h^** | | | | 29135 (60) | | 21300 (70)*** | | 7197 (65)*** | | 15352 (70)*** | |
|  | |  | | |  | |  | |  | |  |

**a** ST elevation myocardial Infarction; **b** Percutaneous Coronary Intervention; **c** Left Ventricular Ejection Fraction; **d** Physical activity level; Low= 0-1 sessions/week; Medium = 2-4 sessions/week and High = 5-7 sessions/week; **e** no smoking during the last month; **f** Health related Quality of Life; **g** estimated glomerular filtration rate; **h** ACE-inhibitors, beta-blocking agent, statins or other lipid-lowering agents and anti-trombogenic agents; **i** There was a small internal dropout in some questions. * 0.01 < P ≤ 0.05 differences between included and non-included. ** 0.001 < P ≤ 0.01 differences between included and non-included. *** P ≤ 0.001 differences between included and non-included.
